# EEG alpha phenotypes: linkage analyses and relation to alcohol dependence in an American Indian community study

**DOI:** 10.1186/1471-2350-11-43

**Published:** 2010-03-18

**Authors:** Cindy L Ehlers, Ian R Gizer, Evelyn Phillips, Kirk C Wilhelmsen

**Affiliations:** 1 From the Department of Molecular and Integrative Neurosciences, The Scripps Research Institute, 10550 North Torrey Pines Rd., SP30-1501, La Jolla, CA 92037, USA; 2 Molecular and Experimental Medicine, The Scripps Research Institute, 10550 North Torrey Pines Rd., SP30-1501, La Jolla, CA 92037, USA; 3 Department of Genetics, University of North Carolina at Chapel Hill, 120 Mason Farm Road Room 5015 Genetic Medicine Building CB 7264, Chapel Hill, NC 27599-7264, USA; 4 Department of Neurology, The Carolina Center for Genome Sciences and the Bowles Center for Alcohol Studies, University of North Carolina, 4109 Neurosciences Research Bldg, CB#7264, Chapel Hill, NC 27599-7264, USA

## Abstract

**Background:**

Evidence for a high degree of heritability of EEG alpha phenotypes has been demonstrated in twin and family studies in a number of populations. However, information on linkage of this phenotype to specific chromosome locations is still limited. This study's aims were to map loci linked to EEG alpha phenotypes and to determine if there was overlap with loci previously mapped for alcohol dependence in an American Indian community at high risk for substance dependence.

**Methods:**

Each participant gave a blood sample and completed a structured diagnostic interview using the Semi Structured Assessment for the Genetics of Alcoholism. Bipolar EEGs were collected and spectral power determined in the alpha (7.5-12.0 Hz) frequency band for two composite scalp locations previously identified by principal components analyses (bilateral fronto-central and bilateral centro-parietal-occipital). Genotypes were determined for a panel of 791 micro-satellite polymorphisms in 410 members of multiplex families using SOLAR.

**Results:**

Sixty percent of this study population had a lifetime diagnosis of alcohol dependence. Analyses of multipoint variance component LOD scores, for the EEG alpha power phenotype, revealed two loci that had a LOD score of 3.0 or above for the fronto-central scalp region on chromosomes 1 and 6. Additionally, 4 locations were identified with LOD scores above 2.0 on chromosomes 4, 11, 14, 16 for the fronto-central location and one on chromosome 2 for the centro-parietal-occipital location.

**Conclusion:**

These results corroborate the importance of regions on chromosome 4 and 6 highlighted in prior segregation studies in this and other populations for alcohol dependence-related phenotypes, as well as other areas that overlap with other substance dependence phenotypes identified in previous linkage studies in other populations. These studies additionally support the construct that EEG alpha recorded from fronto-central scalp areas may represent an important endophenotype associated with alcohol and other substance dependence.

## Background

Although tribes differ with regard to the use of alcohol and drugs, the United States Indian Health Service has cited substance abuse as one of the most urgent health problems facing Native Americans [1-4]. It has been reported that several Native American communities have alcohol dependence rates that are 4-5 times higher than the general U.S. population [5-9]. In one American Indian community, lifetime alcohol dependence rates have been estimated at 65% in men and 54% in women [10]. Although substance dependence has been found to have a heritable component in this Indian community, specific genetic factors and the genes that encode for them have yet to be fully identified [10-16].

Electrophysiological measures are highly heritable phenotypes [17-22] that may aid in linking brain function to the processes involved in the development of substance dependence in the general population [23-25], as well as in Indian populations [10,26,27]. Several features of the resting EEG have been shown to be genetically influenced, and EEG phenotypes based on frequency and amplitude characteristics that may be useful in genetic analyses have been suggested [22,28,29]. EEG patterns appear to remain highly stable over most of an adult's lifespan [30], with variation within an individual studied on two different occasions being not greater than that observed between monozygotic twins [19].

Early studies investigating the variation in the EEG and its potential genetic basis focused on visually observable patterns in the EEG such as low voltage records with an absence of alpha activity or recordings with monomorphic, continuous alpha waves [30,31]. Early genetic studies identified these patterns as being highly heritable, some having an autosomal dominate mode of inheritance, and provided data in a few families for linkage to a marker on chromosome 20q (see [21,30,32,33] for review). More recent studies, using quantitative measures, have consistently demonstrated that EEG alpha activity has perhaps the highest heritability of all the EEG frequencies, and that a moderately high to high proportion of the genetic variance in the EEG is shared among the frequency bands [34-38]. In order to further clarify the mode of genetic transmission on alpha power, Smit and colleagues [34], examined EEG alpha in a large sample of adolescent twins. Their findings confirmed high heritability for this phenotype but additionally suggested that the mode of genetic transmission was due to additive genetic factors that were largely specific. They additionally found that no significant amount of variance in EEG alpha activity could be attributed to unique environmental factors and that all non-genetic variance was most likely due to unreliability in measurement. They therefore concluded that individual differences in alpha activity, in that population, were little influenced by developmental plasticity and/or individual experiences [34].

Several important studies have begun to identify genes associated with certain EEG phenotypes. Low voltage alpha, in females, has been reported to be associated with a genetic variant that leads to low activity of the enzyme that metabolizes dopamine and norepinephrine, catechol-o-methyltransferase (COMT) [39]. Low voltage alpha has also been reported to be linked to the GABAergic system, as an association has been found between the exon 7 variant of the GABA_B _receptor gene and alpha voltage [40,41]. In a study of Plains Indians, a genome scan revealed evidence for linkage to EEG alpha and beta activity on chromosome 5q13-14 near the corticotropin releasing hormone binding protein (*CRH-BP*) [27]. Additionally, in that study, an association of alpha power to *CRH-BP *was found in Plains Indians as well as in a replication sample of 188 Caucasians [27]. These genetic findings provide evidence that EEG measures are promising endophenotypes in the search for genes involved in alcohol dependence.

In particular, several genetic studies have demonstrated that bipolar EEG measures are highly heritable [36], and may be particularly useful endophenotypes for substance dependence. Porjesz and colleagues [42] have also found significant associations between and the GABAergic system and bipolar measures of the human EEG. They found significant genetic linkage between the beta frequency of the EEG and a cluster of GABA_A _receptors genes on chromosome 4p. Additionally, a GABA_A _receptor gene within this cluster was found to be associated with a DSM-IV diagnosis of alcohol dependence [43].

Genetic studies of complex diseases often have advantages when they are conducted in well-defined populations such as Native American tribes living on reservations [44]. This report is part of a larger study exploring risk factors for substance dependence in an American Indian community [5,10,11,13-15,45-57]. We have previously reported that EEG alpha power is highly heritable in this American Indian community (h^2 ^= 0.62 frontal, 0.67 posterior scalp locations). The current study's aims were to: (1) map loci linked to EEG alpha phenotypes, and (2) to determine if there was overlap of the loci identified for alpha phenotypes and loci previously mapped for alcohol dependence in this American Indian community.

## Methods

Participants were recruited from eight geographically contiguous reservations, with a total population of about 3,000 individuals, using a combination of a venue-based method for sampling hard-to-reach populations [58,59], as well as a respondent-driven procedure [60] as previously described [5,13]. The venues for recruitment included: tribal halls and culture centers, health clinics, tribal libraries, and stores on the reservations. A 10-25% rate of refusal was found depending on venue. Refusal rates were higher at tribal libraries and stores than health clinics and tribal halls/culture centers. Transportation from their home to The Scripps Research Institute was provided by the study.

To be included in the study, participants had to be an Indian indigenous to the catchment area, at least 1/16th Native American Heritage (NAH), between the age of 18 and 70 years, and be mobile enough to be transported from his or her home to The Scripps Research Institute (TSRI). Participants were excluded from electrophysiological analyses if they had a history of head trauma or were currently using medications that could bias the EEG recording. The protocol for the study was approved by the Institutional Review Board (IRB) of TSRI, and the Indian Health Council, a tribal review group overseeing health issues for the reservations where recruitment was undertaken.

Potential participants first met individually with research staff to have the study explained and give written informed consent. During a screening period, participants had blood pressure and pulse taken, and completed a questionnaire that was used to gather information on demographics, personal medical history, ethnicity, and drinking history [61]. Participants were asked to refrain from alcohol and drug usage for 24 hours prior to the testing. No individuals with detectable breath alcohol levels were included in the study dataset (n = 3). During the screening period, the study coordinator also noted whether the participant was agitated, tremulous, or diaphoretic and their data were eliminated from subsequent analyses. Each participant also completed an interview with the Semi-Structured Assessment for the Genetics of Alcoholism (SSAGA) and the family history assessment module (FHAM) [62], which was used to make substance use disorder and psychiatric disorder diagnoses according to Diagnostic and Statistical Manual (DSM-III-R)[63] criteria in the probands and their family members [63]. The SSAGA is a semi- structured, poly-diagnostic psychiatric interview that has undergone both reliability and validity testing [62,64]. It has been used in another Native American sample [65,66]. Personnel from the Collaborative Study on the Genetics of Alcoholism (COGA) trained all interviewers. The SSAGA interview includes retrospective lifetime assessments of alcohol use, abuse, and dependence. A research psychiatrist/addiction specialist made all best final diagnoses.

Six channels of bipolar EEG (F3-C3, C3-P3, P3-01 and F4-C4, C4-P4, P4-02, international 10-20 system) were obtained using an electrode cap (impedance < 5 K ohms), as described. Bipolar recordings were obtained for comparison to previous studies in a wide range of ethnic groups [67-70]. A forehead ground electrode was used. An electrode placed left lateral infra-orbitally and referenced to the left earlobe was used to monitor both horizontal and vertical eye movement. Resting EEG was recorded in a temperature and noise controlled room while a participant was comfortably sitting on a chair. Participants were instructed to relax and keep their eyes closed, but to remain awake throughout the EEG recording. Ten to 15 minutes of EEG was collected on paper (Nihon Kohden, high-low pass filters 1-70 Hz) and also digitized for subsequent analyses. EEG records were monitored during all recordings for signs of drowsiness or artifact. Ten minutes of artifact-free, drowsiness-free EEG, as defined by Daly and Pedley [71], was computer analyzed for each channel. Muscle and movement artifact are identified by a computer driven algorithm that identifies epochs with waveforms that are between 0.25 and 0.5 Hz with an amplitude of higher that 100 microvolts squared per Hz (movement artifacts) and waveforms that are between 20 and 50 Hz with amplitudes above 25 microvolts squared per Hz (muscle artifact). These epochs are verified by the user as artifact and are then removed prior to processing. Time of recording with respect to the menstrual cycle was not controlled, as previous studies have demonstrated that the EEG variables under study are not sensitive to time during the cycle [72].

Records were digitized at 128 Hz. The Fourier transform of consecutive four-second epochs (minimum 140) was calculated and the power spectrum produced using an IBM compatible PC with software developed by Ehlers and Havstad [73]. Power density is calculated in microvolts squared per octave, a transformation that expands amplitudes at high frequencies and reduces them at low frequencies, producing a spectrum with less 1/f characteristics [74]. A rectangular window is used. The transformed data were compressed into frequency bands. Mean spectral power density (microvolts squared/octave) in the alpha 7.5-12.0 Hz frequency band was calculated by summing the raw power spectral values within the band, multiplying by a scale factor derived from the calibration signal to produce the total power in the band in microvolts squared, and dividing by the width of the band in octaves. This width is the logarithm of the ratio of the maximum and minimum frequencies in the band, divided by the log of two. The details of the spectral analysis procedures have been previously described [50,73,74].

The data analyses were based on the overall aim that was to map loci linked to EEG alpha power phenotypes and to determine if there was overlap with loci previously mapped for alcohol dependence phenotypes in an American Indian community. To reduce the number of dependent variables in our linkage analyses, a principal component analysis (PCA) was performed over the six bipolar electrode locations for the alpha frequency band. Varimax rotation yielded two components (eigenvalues > 1, range 2.64-2.67). The electrode sites loading on the first factor were the two fronto-central leads (F3-C3, F4-C4) and the electrode sites loading on the second factor were the four more posterior leads (C3-P3, C4-P4, P3-O1, P4-O2) (loadings ranged from 0.64 to 0.93). The two orthogonal factors explained 87% of the variance. Mean power in each band was averaged across the electrode sites within each of the identified components generating a value for mean power (microvolts squared/octave) for each of the regions identified by the PCA for each participant. These values were the dependent variables in the linkage analyses.

One hundred and eighty-one pedigrees containing 1600 individuals were used in the genetic analyses. Of these, 410 individuals have both genotype and phenotype data and 236 additional individuals have only phenotypic data. Sixty-six families have only a single individual with phenotype data. These individuals were included within some analyses to the extent that they contribute information about trait means and variance and the impact of covariates. The family sizes for the remaining families ranged between 4 and 41 subjects (average 12.19 ± 8.19). Eighty-one families were genetically informative. The data includes 142 parent-child, 260 sibling, 53 half sibling, 11 grandparent-grandchild, 235 avuncular, and 240 cousin relative pairs. Only sibling, half-sibling, avuncular and cousin pairs were included as being potentially genetically informative. Many individuals can be linked to a few large extended pedigrees with many founders and complex "loop" structures, which were "broken" to simplify the analysis.

DNA was isolated from whole blood using an automated DNA extraction procedure, genotyping was done as previously described [75]. Genotypes were determined for a panel of 791 autosomal microsatellite polymorphisms [76] using fluorescently labeled PCR primers under conditions recommended by the manufacturer (HD5 version 2.0; Applied Biosystems, Foster City, CA). The HD5 panel set has an average marker-to-marker distance of 4.6 cM, and an average heterozygosity of greater than 77% in a Caucasian population. Allele frequencies were estimated from the entire Mission Indian population with genotype data. Gender and age accounted for greater than 5% of the phenotypic variance for each of the phenotypes. Therefore, age and gender were included as covariates in the analyses.

Genotypes were ultimately determined for 410 subjects. Samples for which less than 90% of genotypes met quality standard were repeated for the entire panel. Ultimately 273,598 genotypes were accepted. Less than 10% of the sample had the majority of the failed genotypes. All available genotypes for all of the autosomal markers were analyzed for each family using PREST [77] to detect sample and pedigree structure errors resulting in the removal of 6 individuals from further analyses. PREST assesses degree of allele sharing and calculates several statistics for each relative pair that are each sensitive difference type of pedigree miss-specification. Pedcheck was used to detect non-Mendelian inheritance patterns [78]. Relevant genotypes were reviewed blind to diagnosis. Very few Mendelian inconsistencies could be resolved by review of electropherograms. Genotypes for the nuclear family were removed for each Mendelian inconsistency. A total of 772 genotypes were removed from linkage analysis because of Mendelian inconsistencies. To further reduce errors, the probability that each genotype is correct was assessed in the context of all other available genotypes using the maximum-likelihood error-checking algorithm implemented in Merlin [79]. Genotypes that had a probability of less than 0.025 of being correct were removed from further consideration. A total of 508 genotypes were removed in this step. Duplicate genotypes were available for a large fraction of the genotype problems detected by Pedcheck and Merlin. In almost all cases these problematic results are reproducible, suggesting somatic mutations, mosaicism or "null alleles" (the failure to amplify the allele from one chromosome resulting in the assumption that an individual is homozygous for the other allele). In our previous experience about 0.5% of microsatellite genotypes using the HD5 panel give reproducible results that are inconsistent with other family genotype data.

Variance component estimate methods were used to calculate LOD scores using SOLAR v2.0.4 [80,81]. Simulation analyses were then conducted in which a genetic locus was simulated under the null hypothesis of no linkage across 50,000 trials to derive nominal p-values for the reported LOD scores [82].

## Results

Four hundred and ten participants' EEG records were available for these analyses. Three hundred sixty-six (60%) of participants met criteria for Alcohol dependence. Demographics of this sample are presented in Table 1. There were no significant differences in the demographics between the participants with EEG records and genotyping available (e.g. the linkage sample, n = 410) and the entire sample of participants in the study with valid SSAGA data (n = 628) but no genotyping and/or EEG data.

**Table 1:** Demographics

	Linkage Sample (n = 410)	Entire Sample (n = 628)
**Gender**	M = 162, F = 248	M = 260, F = 368
**Married**	84	108
**Employed**	188	259
**Income ≥ $20,000 year**	195	323
**Native American Heritage ≥ 50%**	165	255
**Age**	30.1 ± 0.6	30.5 ± 0.5
**Education**	11.6 ± 0.1	11.6 ± 0.1

**Figure 1:**
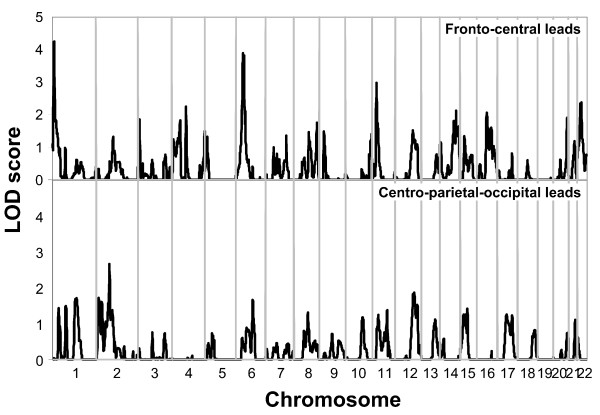
**Multipoint Linkage Analysis for the EEG alpha power in fronto-central leads (upper graph), EEG alpha power in the centro-parieto-occipital leads (lower graph) phenotypes for the entire genome**. Results for each chromosome are aligned end to end with the p terminus on the left. Log of the Odds (LOD) score is plotted on the Y-axis. Vertical lines indicate the boundaries between the chromosomes. The numbers above on the X-axis indicate the chromosome number.

**Figure 2:**
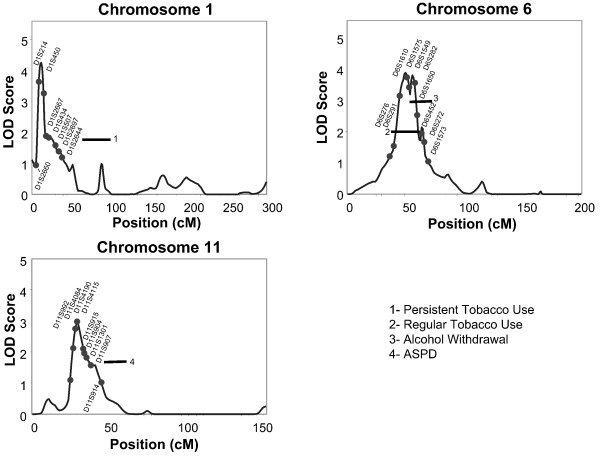
**Multipoint Linkage Analysis for EEG alpha power in fronto-central leads phenotype for chromosomes 1,6,11. Log of the Odds (LOD) score (Y-axis) is plotted for the chromosome location map (in centimorgans (cM), X-axis)**. Nearest markers to the peak are presented within the support interval. Horizontal bars are presented to indicate the support interval and LOD score for other phenotypes where evidence for linkage has been found in this population. ADPD = antisocial personality disorder, regular tobacco = smoking daily for a month or more, persistent tobacco = smoking 10 or more cigarettes a day for more than a year.

Analyses of multipoint variance component LOD scores, for the EEG alpha phenotype, revealed two loci that had a LOD score of 3.0 or above for the fronto-central scalp region on chromosomes 1 and 6. Four additional locations were identified with LOD scores above 2.0 on chromosomes 4, 11, 14, and 16 for the fronto-central location, and one on chromosome 2 for the centro-parietal-occipital location. Figure 1 presents the linkage peaks generated by these analyses across the genome, and figure 2 presents individual chromosome data for the three largest LOD scores of chromosomes 1, 6 and 11. Table 2 presents the peak LOD scores, the closest marker location for the loci identified, empirical p values, and additionally gives information of other findings in the literature for substance related phenotypes observed at or near those locations.

**Table 2:** Genetic Loci for EEG traits in an American Indian community

CHR	Trait	LOC (cM)	LOD	Nearest Marker	Nominal p-value	Supporting References (phenotype)
1	Fronto-central alpha	12	4.25	D1S214/ D1S450	0.00012	Gizer et al., unpublished work (Alcohol Dep)
2	Centro-parietal-occipital alpha	92	2.66	D2S286	0.00314	[15] (ASPD); [85] (Alcohol Dep); [94,95] (Smoking)
4	Fronto-central alpha	98	2.25	D4S2460	0.00318	[13] (Drinking/Dep symptoms); [85] (Alcohol Dep); [86] (Quant Alcohol Phenotype); [87] (Max Drinks); [88,89] (Drinking)
6	Fronto-central alpha	50	3.90	D6S1575	0.00022	[13] (Alcohol Withdrawal); [90,92,93] (Smoking);
11	Fronto-central alpha	30	2.98	D11S4115	0.00118	[15] (ASPD); [96] (Opioid Dep); [97] (Alcohol Dep)
14	Fronto-central alpha	113	2.13	D14S65	0.0039	[51,98] (Cannabis Dep); [99] (Opioid Dep); [100] (Ever Smoked)
16	Fronto-central alpha	69	2.07	D16S415/ D16S3140	0.0051	[101] (Ever quit smoking)
22	Fronto-central alpha	29	2.38	D22S280/ D22S277	0.00174	[27] (Theta EEG); [75] (LR to alcohol); [[88,103], Gizer et al., unpublished work (Alcohol Dep); [102] (Smoking)

Identified loci for EEG alpha phenotypes were found to overlap with regions of interest identified in previous genome scans for alcohol dependence related phenotypes conducted in this Indian population. The area on chromosome 4 that was identified in the present linkage analysis for frontal alpha power, was found previously for an alcohol drinking symptoms severity phenotype as well as for a "persistent smoking" phenotype in this Indian population [13,47]. The area on chromosome 6 that was identified in the present linkage analyses for frontal alpha power was also found previously in a genome scan for an alcohol withdrawal phenotype in this Indian population [13].

## Discussion

Identifying biomarkers of alcohol dependence in Native American populations is important because of the high burden of morbidity and mortality that substance use disorders pose to some Native American groups. As reported previously, Indians in this sample have high rates of alcohol (65% in men 54% in women) and cannabis (32.3%) dependence and antisocial personality disorder (9.8%), but not higher rates of major depressive disorder (8.7%) or anxiety disorders (9.02%) [13,14,48,54,55]. Our finding of high rates of DSM-III-R alcohol dependence is consistent with the findings of investigators working in several other native communities [6-9]. The identification of biomarkers that may represent endophenotypes in Indian populations may aid in genetic studies seeking to identify inherited factors that may contribute to the high rates of substance dependence in these populations.

One set of endophenotypes that may be particularly informative for the genetic analysis of substance dependence and other psychiatric disorders are human electrophysiological measures [e.g. EEG, event-related potentials (ERPs), event-related oscillations (EROs)] [83]. The EEG is a highly heritable, quantitative, biological measure that is less complex and presumably closer to gene function than diagnostic and psychological measures of substance dependence. We have previously reported that the heritability estimates of bipolar EEG power measures in this Indian community sample ranged from 0.16 to 0.67 [50]. These findings were comparable to that reported for the COGA sample by Tang and colleagues [36] who reported heritability estimates from 0.22 to 0.64 for bipolar EEG recordings. In both studies, the highest heritabilities were observed in the alpha and beta frequencies, particularly in posterior areas. Taken together these studies suggests that the EEG is significantly heritable in this Indian population and that heritability estimates for this population appear to be very similar to what has been found in the COGA population.

Several areas of the genome were found to have LOD scores above 2 that appear to be linked to the heritable phenotype of EEG bipolar alpha power in this Indian population. There are two loci for the alpha power phenotype, found in this Indian community study, that were identified in previously published linkage analyses for substance dependence phenotypes in this Indian population [13]. One site was on chromosome 4 at @98 cM (LOD = 2.25) near the ADH gene cluster. Bivariate linkage analysis was conducted at this site on chromosome 4 for persistent tobacco use and an alcohol drinking severity phenotype also previously identified at this site. The maximum LOD score for the bivariate analysis for this region was 3.4 [47]. This site has also been found to be linked to alcohol dependence [84,85] a quantitative alcohol-related phenotype [86], and maximum number of drinks [87] in the COGA population. It has additionally been identified with alcohol drinking phenotypes in a cohort selected for smoking behavior [88] and in the Irish Affected Sib-pair study [89]. The second region identified in the present genome scan for an alpha EEG power phenotype was a region on chromosome 6 at @50 cM (LOD = 3.9) that was found previously in this Indian community sample for an alcohol withdrawal phenotype [13]. A region near this site appears to have been found to be linked to several smoking phenotypes [90-93]. The gene for the *GRM4 *glutamate receptor is also found in this region of chromosome 6. Thus it appears that these areas on chromosomes 4 and 6 may harbor genes for both the EEG alpha phenotype as well as a number of substance related traits observed in this Indian population as well as in other population samples.

Six other areas of the genome provided LOD scores suggesting evidence for linkage to the bipolar alpha power phenotype in this Indian population. One site was on chromosome 1 at @12 cM with a LOD score of 4.25. The same locus was previously identified in a genome scan for alcohol dependence (Gizer and colleagues, unpublished work). This site harbors a number of genes of relevance including: the GABA delta receptor gene *GABRD*, an aldehyde dehydrogenase gene *ALDH4A1*, a serotonin receptor gene *HTR1D*, a cannabinoid receptor gene *CNR2*, and a circadian rhythm gene *PER3*. A second site was on chromosome 2 @92 cM (LOD = 2.66). This site is near a location (99 cM) that has been reported previously to be linked to alcohol dependence [85,94] and habitual smoking [95] in the COGA sample. This site on chromosome 2 also appears to be within 10 cM of a site reported previously for an antisocial personality disorder phenotype in this Indian population [15]. Another site was identified on chromosome 11 for the EEG alpha power phenotype in this Indian population at @30 cM with a LOD score of 2.98. This site appears to be within 15 cM of a site identified in a genome scan for opioid dependence in a sample of small nuclear families recruited from 4 sites [96], and also may overlap with a site identified for alcohol dependence in a SW Indian tribe [97]. Several genes of interest are found within this site including: a glycine transporter *SLC6A5*, brain-derived neruotrophic factor *BDNF*, and a serotonin precusor tryptophan hydroxlyase *TPH1*. Another site identified in the present genome scan was on chromosome 14 at @113 cM (LOD = 2.1). This site was found in two genome scans for cannabis dependence, one in this Indian population [51] and one in the COGA study [98]. This site on chromosome 14 was also found to be within 20 cM of a site identified by Lachman and colleagues [99] for opioid dependence in a mixed racial population and for an "ever smoked" phenotype in the COGA sample [100]. On chromosome 16 a site was identified at @70 cM with a LOD score of 2.06 that was also found in a genome scan for an "ever quit smoking phenotype" [101]. The concordance between studies in identifying several loci in the genome that are associated with substance dependence phenotypes and alpha power further suggests that the search for candidate genes within these locations may be productive in identifying some general mechanisms that may underlie variation in both phenotypes.

There have been two published studies that have reported linkage findings specifically for EEG phenotypes. One study was conducted using the COGA sample [42]. In that study evidence was found for linkage and linkage disequilibrium at a GABA_A _receptor gene on chromosome 4 (at @56 cM) for the beta frequencies of the EEG [42]. In another genome scan, conducted in Plains American Indians, a site within 10-15 cM of this site on chromosome 4 was also identified for theta and alpha EEG power [27]. This site was not identified in the present study. Another site identified in the study of EEG phenotypes in Plains Indians was on chromosome 5 in a region between 53 and 114 cM. This site appears to be linked to theta, alpha and beta power in the Plains Indian population [27]. Although this site was not identified in the present genome scan for EEG alpha phenotypes it was found in a previous genome scan for an "alcohol craving" phenotype in the present Indian population (LOD score = 4.5) [11]. One site identified in the present study on chromosome 22 at @29 cM (LOD = 2.4) for the alpha power phenotype was also found in the genome scan in Plains Indians for a theta EEG phenotype [27]. This general region has also been identified in genome scans for a number of other substance abuse related phenotypes such as smoking [102], alcohol dependence [[16,103], Gizer et al., unpublished work) and level of response to alcohol [75].

Interestingly 7 of the eight loci with LOD scores above 2 identified in the present study were from the fronto-central scalp location. The important of frontal cortical brain areas for a variety of impulsive behaviors including substance use disorders has been documented in a number of human and animal studies [104,105]. Disruption of orbitofrontal cortex laterality has been demonstrated in offspring from multiplex alcohol dependence families [106]. Smaller prefrontal cortices have also been shown to be associated with early-onset drinking in individuals with co-morbid mental disorders [107]. White matter microstructure deficits have also been identified several right hemisphere tracts connecting prefrontal and limbic systems in abstinent alcoholic men [108]. Taken together these studies suggest that heritable factors may contribute to some aspects of the functioning of frontal lobe areas that have been associated with substance use disorders.

## Conclusion

In conclusion, two loci that had a LOD score of 3.0 or above for an EEG alpha power in fronto-central areas phenotype were found on chromosomes 1,6. Additionally, 4 loci were identified with LOD scores above 2.0 on chromosomes 4, 11, 14, and 16. One loci was identified on chromosome 2 for the centro-parieto-occipital region. These results corroborate the importance of regions on chromosome 4 and 6 highlighted in prior segregation studies in this and other populations for alcohol dependence-related phenotypes, as well as other areas that overlap with other substance dependence phenotypes identified in previous linkage studies in other populations. However the results of this study should be interpreted in the context of several limitations. First, the findings may not generalize to other Native Americans or represent all Indians within this population. Second, only retrospective and cross-sectional data on alcohol use disorders were assessed. Despite these limitations, this report represents an important step in an ongoing investigation to determine genetic and environmental factors associated with substance use and use disorders in this high risk and understudied ethnic group.

## List of Abbreviations

CNS: Central Nervous System; COGA: Collaborative study on the genetics of alcoholism; DNA: Deoxyribonucleic Acid; DSM-III-R: Diagnostic and Statistical Manual; EEG: electroencephalogram; ERPs: event-related potentials; EROs: event-related oscillations; FHAM: family history assessment module; Hz: cycles/second; IRB: Institutional review board; LOD: log of the odds ratio; NAH: Native American heritage; PCA: principal component analysis; PCR: polymerase chain reaction; SSAGA: semi-structured assessment for the genetics; SOLAR: Sequential Oligogenic Linkage Analysis Routines.

## Competing interests

The authors declare that they have no competing interests.

## Authors' contributions

Cindy L Ehlers contributed to the recruitment, collection and analysis of the clinical and genetic data on the subjects. Evelyn Phillips collected all EEG data and conducted power spectral analyses. Kirk C. Wilhelmsen contributed to the genotyping. Ian Gizer conducted linkage analyses. All authors contributed to writing the paper.

## Pre-publication history

The pre-publication history for this paper can be accessed here:

http://www.biomedcentral.com/1471-2350/11/43/prepub
